# Peripheral Blood Monocytes as Adult Stem Cells: Molecular Characterization and Improvements in Culture Conditions to Enhance Stem Cell Features and Proliferative Potential 

**DOI:** 10.1155/2016/7132751

**Published:** 2015-12-20

**Authors:** Hendrik Ungefroren, Ayman Hyder, Maren Schulze, Karim M. Fawzy El-Sayed, Evelin Grage-Griebenow, Andreas K. Nussler, Fred Fändrich

**Affiliations:** ^1^First Department of Medicine, UKSH, Campus Lübeck, Ratzeburger Allee 160, 23538 Lübeck, Germany; ^2^Clinic for Applied Cellular Medicine, UKSH, Campus Kiel, Arnold-Heller Strasse 3, Haus 18, 24105 Kiel, Germany; ^3^Faculty of Science, Damietta University, Damietta 34517, Egypt; ^4^Department of General, Visceral and Transplantation Surgery, University Hospital Essen, Hufelandstrasse 55, 45147 Essen, Germany; ^5^Clinic for Conservative Dentistry and Periodontology, School of Dental Medicine, UKSH, Campus Kiel, Arnold-Heller Strasse 3, Haus 26, 24105 Kiel, Germany; ^6^Oral Medicine and Periodontology Department, Faculty of Oral and Dental Medicine, Cairo University, 1 Mathaf El Manial Street, Giza, Egypt; ^7^BG Unfallklinik Tübingen, Eberhard Karls Universität Tübingen, Schnarrenbergstraße 95, 72076 Tübingen, Germany

## Abstract

Adult stem or programmable cells hold great promise in diseases in which damaged or nonfunctional cells need to be replaced. We have recently demonstrated that peripheral blood monocytes can be differentiated *in vitro* into cells resembling specialized cell types like hepatocytes and pancreatic beta cells. During phenotypic conversion, the monocytes downregulate monocyte/macrophage differentiation markers, being indicative of partial dedifferentiation, and are partially reprogrammed to acquire a state of plasticity along with expression of various markers of pluripotency and resumption of mitosis. Upregulation of stem cell markers and mitotic activity in the cultures was shown to be controlled by autocrine production/secretion of activin A and transforming growth factor-beta (TGF-*β*). These reprogrammed monocyte derivatives were termed “programmable cells of monocytic origin” (PCMO). Current efforts focus on establishing culture conditions that increase both the plasticity and proliferation potential of PCMO in order to be able to generate large amounts of blood-derived cells suitable for both autologous and allogeneic therapies.

## 1. Introduction

Adult stem or programmable cells represent a promising alternative to embryonic stem cells in regenerative medicine and in tissue engineering [1]. Although not considered a classical adult stem cell, monocytes have been shown to be capable of acquiring stem cell-like properties [2–5]. Peripheral blood monocytes have some practical advantages over other types of adult stem/progenitor cells when they are used for clinical purposes: (1) they can be retrieved from a readily accessible body compartment by a low-invasive procedure or are incurred as waste products in blood donations; (2) they can be readily maintained in culture; (3) they have a low risk of tumorigenicity due to their limited proliferative capacity and the lack of* telomerase reverse transcriptase* (hTERT) [6]; (4) they can be applied to patients in both an autologous and an allogeneic setting, obviating the need for immunosuppression. Autologous cell material for transplantation may otherwise only be derived from adult stem cell populations such as bone marrow-derived stem cells or from human embryonic stem cells generated by somatic cell nuclear transfer or from cells with induced pluripotency (iPS). However, all these potential cell sources suffer from biological, economic, and/or ethical drawbacks [7].

Serious disadvantages of using monocytes are their limited number in the blood circulation and their low proliferation potential* in vitro*. For transplantation purposes, it is necessary to increase cell yields by* in vitro* expansion. Another obstacle is the monocytes' varying differentiation potential into specialized cell types which is largely donor-dependent. To be clinically relevant, conditions should be optimized towards the production of large amounts of cells from one single donor. Therefore, the main goal is to enhance the cells' proliferation potential during culture while at the same time maintaining or even improving their differentiation potential towards the desired cell type. In the course of this paper, we provide an overview on the molecular events during the dedifferentiation phase, for example, when the cells acquire their stem cell-like characteristics, and subsequently discuss various strategies that have been showing promise to increase cell numbers during* in vitro* culture.

## 2. Macrophage Phenotypic and Functional Heterogeneity and Monocyte Plasticity

The circulating monocyte is a very versatile progenitor cell that gives rise to diverse cell types. It is generated from hematopoietic stem cells via the common myeloid progenitor (CMP) and the granulocyte/monocyte progenitor which represents the precursor populations for monoblasts. Monoblasts are the earliest form committed to becoming monocytes and their progeny emigrates from the bone marrow into the peripheral blood. When not recruited to inflammatory lesions, peripheral blood monocytes are capable of undergoing maturation into several types of tissue-resident macrophages (reviewed in [8]) such as resting tissue macrophages, Kupffer cells, Langerhans cells of the skin, dendritic cells, microglia, osteoclasts, and endothelial cells. When appropriately stimulated, monocytes will migrate to sites of inflammation and extravasate from the circulation into the tissues, acquiring the characteristics of an activated macrophage with an inflammatory phenotype. In an acute inflammatory response, this usually entails production of inflammatory cytokines, antimicrobial oxidative radicals, tissue-debriding proteinases, and an elevated phagocytic activity. Once the wound is cleared of inflammatory debris, macrophages contribute to the process of wound resolution, promoting angiogenesis, matrix production, and cell proliferation. This functional switch to an alternatively activated, anti-inflammatory, and regeneration-promoting phenotype appears to be initiated by phagocytosis of apoptotic cells and to be regulated by a variety of tissue-derived cytokines, hormones, and metabolites [9]. In certain carcinomas, macrophages can be recruited into this tissue to adopt a specific phenotype that eventually promotes tumor progression. These tumor-associated macrophages (TAMs) are chronically polarized to exhibit activities that support tumor growth and metastasis, suppress adaptive immune responses, and hence resemble an alternatively activated type [9]. How the functional plasticity of monocytes/macrophages is generated is currently a matter of debate [9].

Functional heterogeneity of macrophages may depend on the differentiation of functional sublineages, or alternatively macrophages are functionally plastic cells which are capable of altering their functional activities progressively in response to changing signals generated in their microenvironment (functional plasticity hypothesis). The functional plasticity and regenerative potential of monocytes/macrophages may be much greater than what is previously thought. Several cultured human cell populations that originate from circulating monocytes have the capacity to differentiate into nonphagocytic pluripotent stem cell-like cells such as pluripotent stem cells (PSCs) [2], MOMC [3, 5], CD14+CD34^low^KDR+ subset [10], and PCMO [4, 11, 12].

## 3. Differentiation Potential of PCMO and Molecular Evidence for Monocytes as Potential Stem Cells

Recently, we have developed a protocol to induce from monocytes by* in vitro* culture an apparently more plastic derivative, which we named “programmable cells of monocytic origin” (PCMO). These cells following a 4–6-day treatment with macrophage colony-stimulating factor (M-CSF), interleukin-3 (IL-3), and human serum were susceptible following exposure to appropriate induction media to differentiate into cells with endothelial characteristics, chondrocytes, and osteoblasts/osteocytes (manuscript in preparation). A study by Yang and coworkers suggested* in situ* osteogenic differentiation of and bone formation by transplanted human PBMNCs [13]. We have also shown earlier that PCMO generated from human peripheral blood or from either blood or spleen of a nonhuman primate can be converted to insulin-producing cells [4]. However, our focus was on PCMO-derived hepatocyte-like cells (so-called NeoHepatocytes), which express various hepatocyte markers and exhibit hepatocyte-specific metabolic functions* in vitro* and* in vivo* [4, 11]. Intriguingly, NeoHepatocytes were able to improve survival in a rat model of acute liver failure [14] and monocyte-derived hepatocyte-like cells even showed promise in the treatment of HBV-related decompensated liver cirrhosis [15]. A more general loss of the monocyte/macrophage phenotype would thus be in favor of a dedifferentiation process and would lend support to our contention that PCMO represent cells which have reverted to a more primitive progenitor with less restricted differentiation potential. In line with this, it was found that in response to the specific culture conditions monocytes silence the monopoietic marker genes* PRDMI* and* ICSBP* while maintaining expression of the lineage-specific transcription factors* PU.1* [4]. Even more informative was the pattern of Krüppel-like factor 4 (Klf4), a nuclear factor of monocyte → macrophage differentiation which is expressed in a monocyte-restricted and stage-specific pattern during myelopoiesis and promotes inflammatory monocyte differentiation [16].* Klf4* was rapidly silenced following exposure of monocytes to PCMO culture conditions and transcript levels remained low until day 6 after which they became undetectable. Other markers associated with specialized functions, for example, those involved in sensing and killing of invading microorganisms such as CD14, toll-like receptors (TLRs 2, 4, 7, and 9), and two subunits of the reactive oxygen producing enzyme NAD(P)H oxidase (Nox4 and p47^phox^), respectively, were downregulated although with different kinetics [6]. Expression of TLRs and Nox4 was dramatically decreased at the transcriptional level on day 6 of culture, while expression of p47^phox^ remained unaltered at the mRNA level but appeared to be downregulated at the protein level starting from day 2 in PCMO medium [6]. A list of markers that are downregulated during the monocyte → PCMO conversion is provided in Table 1.

### 3.1. Expression and Reactivation of Pluripotency Markers during PCMO Generation

Despite the well-documented broad differentiation potential of monocytes, surprisingly little is known about the mechanisms which allow them to maintain an uncommitted precursor state. We therefore wondered whether the mechanisms underlying the phenotypic plasticity of ESCs also operate in PCMO. In this respect, we provided evidence at the mRNA and protein level that PCMO share in common several markers of ESCs such as OCT4 (also termed POU5f, including OCT4A, the isoform associated with pluripotency) and NANOG [6]. Both proteins can function as activators of self-renewal and pluripotency genes and as repressors of lineage commitment genes. Interestingly, the endogenous* OCT4* and* NANOG* genes appear to be reactivated and the kinetics corresponded well with the time-dependent pattern of sensitivity towards hepatocytic differentiation [6]. Other genes implicated in maintenance of self-renewal and pluripotency and expressed in PCMO include* growth and differentiation factor 3* (*GDF3*),* DPPA3*/*STELLA*/*PGC7*,* ABCG2*,* Connexin-43*,* NCAM*,* DNMT3b*,* UTF*,* BMP2*,* CRIPTO*/*TDGF1*,* E-CADHERIN*, and* CD105*. Other genes like* SOX2*,* GABRB3*,* NODAL*,* LEFTY B*, and* hTERT* were negative in PCMO. The data indicate that critical pluripotency regulators (e.g., OCT4 and NANOG) and possibly other factors of the pluripotency network, such as Myc (see Table 1), are reactivated in PCMO and likely control their plastic behavior. Indeed, PCMO appear to resemble in some aspects partially reprogrammed cell lines [17] in that they reactivate genes related to stem cell renewal and maintenance (e.g.,* MYC*) and pluripotency (*OCT4*,* NANOG*). These results show that in appropriate growth factor environment circulating monocytes can, at least partially, be reprogrammed without exogenous introduction of pluripotency factors.

### 3.2. TGF-*β*/Activin Signaling and Enhancement of Pluripotency Marker Expression

In ES cells, maintenance of and exit from the undifferentiated stage are primarily controlled by members of the TGF-*β*/activin family of growth factors through regulation of the pluripotency-associated transcription factors OCT4 and NANOG [18, 19]. There is also evidence for a role of TGF-*β*/activin-related factors in regulating stemness of monocytes/PCMO. We have shown recently that TGF-*β* and its receptors, TGF-*β* type II receptor and activin receptor-like kinase 5 (ALK5), are expressed in PCMO. Moreover, we found that TGF-*β* is secreted by PCMO into the culture supernatant as determined by ELISA, with levels of TGF-*β*1 declining during monocyte conversion to PCMO. In contrast, levels of activin A (the *β*_A-_ *β*_A_ homodimer) in the culture supernatants* increased* until day 4 of culture. Interestingly, PCMO express not only the activin A ligand but also the receptors ActRIIA and ALK4, both of which were upregulated until day 4 of culture, suggesting enhanced responsiveness of the cells to (autocrine) activin stimulation. Reciprocal autocrine signaling by TGF-*β* and activin in the cultures was confirmed by the demonstration that C-terminal phosphorylation/activation of Smad3, the primary target of ALK5, declined until day 4 of culture, while activation of Smad2, the primary target of ALK4, rose over the same time period. We confirmed sensitivity of PCMO to activin signaling by showing that recombinant activins (A, B, and AB) induced C-terminal phosphorylation of Smad2 but not Smad3.

### 3.3. Epigenetic Changes during PCMO Generation

The methylation of histones plays a crucial role in epigenetic regulation of gene expression during mammalian development. In general, transcribed genes are associated with trimethylation at Lys-4 of histone H3 (me-H3(K4)) [20], whereas many silenced genes are associated with H3(K27) trimethylation [21]. Interestingly, the induction of both* NANOG* and* OCT4* coincided with transient changes in histone modifications indicative of transcriptional (re)activation [6]. In undifferentiated cells, the* OCT4* promoter is packaged with nucleosomes that contain markers of active chromatin, namely, histone H3 highly acetylated at Lys-9 and Lys-14, and me-H3(K4) [22]. We noted a transient rise in global me-H3(K4) in PCMO cultures that closely mirrored the time course of* OCT4* transcriptional activity. The methylation of the* OCT4* promoter remained high during various stages of PCMO generation, as revealed by bisulfite conversion and pyrosequencing at specific CpG islands in the* OCT4* distal enhancer [6], suggesting that promoter demethylation was not the underlying mechanism of* OCT4* induction. Preliminary data indicate that treatment of PCMO with 5-azacytidine can increase their plasticity. We are currently studying whether this is reflected also in the upregulation of pluripotency-determining genes.

## 4. Culture Conditions Favoring Stem Cell Features of Monocyte

We are currently pursuing three strategies to enhance the stem cell character of PCMO under the assumption that a more stem cell-like progenitor will impact the phenotype of the desired differentiated cell type: (1) avoidance of activating/differentiation stimuli (proinflammatory agents and bacterial components) which might prevent proper dedifferentiation of monocytes, (2) comparison of PCMO plasticity and proliferative activity of PCMO under suspended and adherent growth conditions, and (3) enhancement of PCMO plasticity/pluripotency marker expression by modulation of activin and TGF-*β* signaling.

### 4.1. Avoidance of Activating/Differentiation Stimuli Which Might Prevent Proper Dedifferentiation of Monocytes

In order to prevent activation to proinflammatory macrophages, it seems mandatory to deplete the medium of the cells of any potential proinflammatory agents, bacterial components, or foreign antigens that cause activation of monocytes in the course of an immune reaction. Our data clearly demonstrate that the use of autologous serum reduced initial macrophage activation in PCMO cultures and subsequently improved both yield and function of differentiated NeoHepatocytes. An autologous approach might also be useful in other stem cell preparation processes where cell activation during generation shall be kept to a minimum.

### 4.2. Comparison of PCMO Plasticity of PCMO under Suspended and Adherent Growth Conditions

Adherence of primary peripheral blood monocytes is essential for the differentiation into macrophages. On the other side, PCMO development must be accompanied by keeping a state of* de*differentiation. To keep cells in* de*differentiated state, it is theoretically logic to culture them in suspension to prevent differentiation into macrophages from occurring. The switch from suspended to adherent growth which was associated with changes in cell morphology [4] also affected proteins involved in cell adhesion and cytoskeletal regulation such as p60^Src^ in *β*-actin and E-cadherin (which all increased) over the 4–6-day culture period [6]. Together, these data suggest that monocytes during their conversion to PCMO undergo partial* de*differentiation. The expressions of the stem cell marker genes* OCT4* and* NANOG* were higher in adherent than in suspended cells.

The comparison of the resulting differentiated NeoHepatocytes, which originated from adherent and suspended PCMO, showed mixed results. Cells in suspension resulted in NeoHepatocytes with higher levels of CYP1A1/2, CYP2D6, and UDPG and higher urea metabolism, while adherent PCMO resulted in NeoHepatocytes with higher SRB-1 and glucose metabolism. These hepatocyte-specific assays may indicate an independent regulation of PCMO plasticity and proliferation.

### 4.3. Enhancement of PCMO Plasticity/Pluripotency Marker Expression by Modulation of Activin and TGF-*β* Signaling

We have recently shown that during PCMO generation pluripotency marker expression is controlled positively by activin/Smad2 and negatively by TGF-*β*/Smad3 signaling. Specifically, inhibition of autocrine activin signaling by the activin-binding protein follistatin reduced both Smad2 activation and OCT4A/NANOG upregulation. It can be concluded that treatment of PCMO with activin(s) would enhance OCT4A/NANOG expression and pluripotency. Conversely, inhibition of autocrine TGF-*β* signaling by anti-TGF-*β* antibody reduced Smad3 activation and moderately enhanced OCT4A/NANOG expression arguing for TGF-*β* inhibition as an additional or complementary strategy to increase stemness in PCMO. Several TGF-*β* pathway inhibitors (that should not cross-inhibit activin signaling such as the commonly used ALK4/5 small molecule inhibitor SB431542) are available for this purpose [23].

## 5. Strategies to Enhance the Proliferation Potential of PCMO

As mentioned above, expansion and differentiation conditions must be optimized towards the production of large amounts of PCMO from one single donor. We have previously shown that monocytes resume proliferation under PCMO culture conditions [6]; however, the percentage of cells that reentered mitosis was still low. Apart from the use of autologous serum which improved the yield of differentiated NeoHepatocytes by increasing cell numbers during the PCMO stage [24], we are currently pursuing three other strategies to achieve this: (1) enhancement of proliferation following adhesion culture, (2) coculture of PCMO with lymphocytes, and (3) addition of mitogenic growth factors.

### 5.1. Comparison of PCMO Proliferation under Suspended and Adherent Growth Conditions

We have compared the proliferative activities of PCMO cultured in suspension* versus* adherence. Proliferation was studied by immunofluorescence for Ki67 that marks the development from G1- to S-phase in the cell cycle. In contrast to our expectations, the data showed that proliferation of PCMO was* higher* in adherently growing than in suspended cultures as revealed by the appearance of a subset of Ki67-positive monocytes and downregulation of p21^WAF1^ [25].

### 5.2. Enhancement of Proliferation following Coculture of PCMO with Autologous Lymphocytes

**Figure 1:**
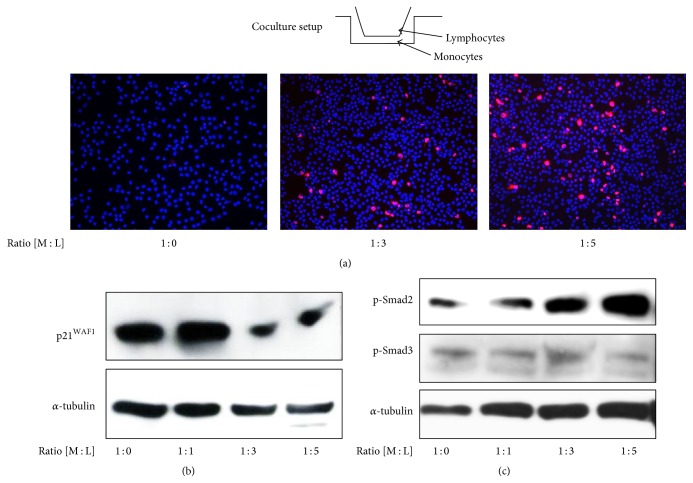
Coculture with lymphocytes increases the fraction of mitotically active PCMO. (a) Adherently growing peripheral blood monocytes isolated by elutriation were indirectly cocultured with increasing numbers of autologous lymphocytes in transwell inserts (top, pore size 0.4 *μ*m). Ratios of monocytes (M) : lymphocytes (L) are indicated below the images. After 4 days, cultured cells were fixed and double-stained with an antibody to Ki67 (magenta) and DAPI (blue) as control. The procedure of elutriation was described previously [25]. (b) Detection of p21^WAF1^ expression in elutriated monocytes cocultured with lymphocytes at different ratios. After coculture, monocytes were lysed and analyzed for expression of p21^WAF1^ by immunoblotting. The housekeeping protein *α*-tubulin served as a loading control. (c) As in (b), except that the immunoblots were probed with antibodies to phospho-Smad2 (p-Smad2) and phospho-Smad3 (p-Smad3). Signal strengths of Smad3 and Smad2 should be assessed relative to those for *α*-tubulin.

The ultimate goal is to enhance the cells' proliferation potential during PCMO culture without impairing their differentiation potential and to further enhance the differentiation potential without compromising their proliferative activity. We observed that PCMO proliferation was much higher in mixed cultures (PBMC “contaminated” with autologous lymphocytes) than in pure cultures of monocytes purified by elutriation, indicating that direct cell-cell interactions or factors secreted by lymphocytes are interacting with PCMO to increase their proliferation. In order to clarify the role of autologous lymphocytes in PCMO proliferation, elutriated monocytes were cocultured with increasing numbers of lymphocytes from the same donor in separated inserts with a pore size of 0.4 *μ*m that allow for the transfer of micromolecules but not of cells (Figure 1(a), top). Our results showed an increase in the fraction of Ki67-positive monocytes that was proportional to the number of cocultured lymphocytes (expressed by the lymphocyte : monocyte ratio, Figure 1). The increase in mitotically active monocytes was accompanied by a downregulation of the cell cycle inhibitor p21^WAF1^ at monocyte : lymphocyte ratios greater than 1 : 1 (Figure 1(b)) and likely reflects a derepression from growth arrest. Since p21^WAF1^ is induced by TGF-*β*/Smad3 signaling and TGF-*β*1 is present in supernatants of PCMO cultures [25], we addressed the question as to whether lymphocytes may inhibit TGF-*β* signaling activity by monitoring the activation status of Smad3 (and Smad2 as control) by phosphoimmunoblotting. In cocultured monocytes, a decrease in phosphorylation of Smad3 was observed relative to monocultured monocytes, while activation of Smad2 was only marginally affected when assessed relative to the levels of the housekeeping protein *α*-tubulin (Figure 1(c)). These results suggest that autologous lymphocytes enhance the proliferation of monocytes by suppressing the growth-inhibitory TGF-*β*/Smad3/p21^WAF1^ axis.

### 5.3. Enhancement of Proliferation following Addition of Mitogenic Growth Factors

#### 5.3.1. EGF/HB-EGF

During earlier studies we observed activation of extracellular signal-regulated kinase (ERK)1 in monocytes with its activity peaking on days 3-4 of PCMO culture [4]. Since the MEK/ERK pathway is activated prominently by epidermal growth factor (EGF), which is known to induce proliferation in many types of cells and its receptor is overexpressed in proliferative cells [26], we evaluated possible direct effects of EGF on PCMO proliferation. Heparin-binding epidermal growth factor (HB-EGF), 20–22 kDa glycoprotein from the EGF family, was also reported to have proliferative effects and to be a potent mitogen for many cell types [27]. Human peripheral blood monocytes were reported to express a functional EGF receptor (EGFR) [28, 29], while the EGF receptors c-erbB2, c-erbB3, and c-erbB4 have not been studied. However, a link between EGF or HB-EGF and proliferation in monocytes has not yet been reported.

EGF and HB-EGF enhanced cell proliferation of PCMO as demonstrated by increased expression of cycle control genes (ABL, ANAPC2, CDC2, CDK4, and CDK6), an increase in phosphorylation of the retinoblastoma protein (Rb → pRb → ppRb), and increased PCMO cell numbers after stimulation with EGF or HB-EGF [30]. EGF also raised the number of monocytes expressing the proliferation marker Ki67. PCMO expressed the EGF receptors EGFR (ERBB1) and ERBB3, and expression of both increased during PCMO generation. Phosphoimmunoblotting of PCMO indicated that both EGF and HB-EGF activated MEK-1/2 and ERK1/2 in a concentration-dependent fashion with the effect of EGF being more prominent [30]. EGF treatment further decreased expression of p47^phox^ and increased that of NANOG indicating enhanced* de*differentiation and pluripotency, respectively. Treatment with both EGF and HB-EGF resulted in NeoHepatocytes with improved functional parameters [30]. The results suggested that the addition of EGF or HB-EGF to PCMO differentiation medium superactivates MEK/ERK signaling which then increases proliferation of PCMO and functional differentiation of PCMO-derived NeoHepatocytes.

#### 5.3.2. TGF-*β*/Activin

A screening for agents that stimulate PCMO proliferation resulted in the identification of the ALK4/5/7 inhibitor SB431542 [31] as an agent that increased the total number of PCMO, probably by preventing the cytostatic function of TGF-*β* on PCMO. Inhibition of autocrine TGF-*β* signaling by either SB431542 or anti-TGF-*β* antibody reduced Smad3 activation and strongly increased the number of Ki67-positive cells. Relief from growth inhibition is primarily the result of reduced TGF-*β*/Smad3 and, to a lesser extent, activin/Smad2 signaling [25]. Inhibition of TGF-*β* receptors during PCMO culture seems to be a suitable tool to further expand PCMO in order to maximize cell yield for future transplantation purposes. However, since SB431542 also inhibits ALK4 and ALK7 and thus activin signaling, care must be taken that this function is not at the expense of a decrease in pluripotency. More specific inhibitors of TGF-*β* signaling need to be employed to avoid this problem [23].

## 6. Concluding Remarks

Within the last decade, we and others have contributed to the realization that monocytes are extremely versatile and plastic cells and that this feature might be exploited* in vitro* to utilize these cells as stem cell-like cells in regenerative medicine. Various cell preparations derived from peripheral blood monocytes have been shown to express markers of pluripotency/ESCs such as OCT4 and NANOG, although their stem cell function has not been demonstrated unequivocally. On a larger scale, similarities were found between transcriptomic profiles of macrophages and murine undifferentiated ESCs but not differentiated stem cells [32]. Moreover, the similarity of monocytes with adult stem cells is also evident from the observation that efficient reprogramming of adult (neural) stem cells to monocytes can be achieved by ectopic expression of only a single gene, namely, PU.1 [33]. PCMO display several features of partially reprogrammed cell lines such as partial reactivation of genes related to stem cell renewal and maintenance (such as* MYC*) and some pluripotency genes (*OCT4* and* NANOG*) and incomplete repression of lineage-specifying transcription factors (such as PU.1). Another feature of stable partially reprogrammed cell lines is DNA hypermethylation at pluripotency-related loci. Based on these observations, it may be possible to further enhance the stem cell character of PCMO and eventually induce a pluripotent state through genetic complementation of* SOX2*, silencing of* PU.1*, and/or treatment of PCMO with small-molecule compounds that modify chromatin or enhance the action of pluripotency factors. It should be mentioned, however, that there is the possibility that the reexpression of* OCT4* in monocytes/PCMO in response to MCSF/IL-3/serum exposure does not reflect a physiological mechanism; nevertheless, it may result in a therapeutically relevant cell type. Interestingly, we observed some similarities in marker expression between PCMO and alternatively activated macrophages such as AMAC-1, FoxP3, and IDO (HU, unpublished). Since M2-polarized macrophages are known to fulfill regenerative functions following tissue damage in the course of an inflammatory process, they not only may contribute to the healing process by secretion of cytokines but also may themselves act as stem cell cells to restore tissue-specific cells. In this respect and in light of our data that autologous serum reduced initial macrophage activation in PCMO and improved both yield and function of differentiated NeoHepatocytes (see above), it would be interesting to compare PCMO with classically activated, alternatively activated, or deactivated macrophages for their expression of pluripotency-determining genes and their multipotency. We believe that both phenotype and metabolic function of PCMO-derived specialized cell types, for example, NeoHepatocytes, can be improved not only by optimizing the conditions for cell-type specific differentiation but also by enhancing even more effectively the PCMO plasticity through the above mentioned strategies.

## Figures and Tables

**Table 1:** List of specific genes and their mode of regulation during the monocyte → PCMO conversion.

Downregulated genes	Upregulated genes
PRDMI	OCT4 (A isoform)
ICSBP	NANOG
KLF4	Myc
CD14	
TLRs (2, 4, 7, 9)	
Nox4	
p47^phox^	
